# Analysis of population structure and genetic diversity of Iranian Wild *Salicornia* (*Salicornia iranica* Akhani) population

**DOI:** 10.1186/s43141-022-00337-0

**Published:** 2022-04-21

**Authors:** Mohammad Aghaei, Abbas Hassani, Hosein Nazemiyeh, Babak Abdollahi Mandoulkani, Mohammad Saadatian

**Affiliations:** 1 Department of Horticulture, Faculty of Agriculture, Urmia University, Urmia, Iran; 2 Pharmaceutical Microtechnology Research Center, Faculty of Pharmacy, Tabriz University of Medical Sciences, Tabriz, Iran; 3 Department of Plant Breeding and Biotechnology, Faculty of Agriculture, Urmia University, Urmia, Iran; 4 General Science Department, Faculty of Education, Soran University, Soran, Erbil, Iraq

**Keywords:** Genetic distance, Cluster analysis, ISSR, Morphological traits, *Salicornia*

## Abstract

**Background:**

*Salicornia* is a halophyte plant capable of being irrigated with seawater, which can be used as an alternative food. Given this, it is necessary to study the potentials of this plant’s morphological diversity in the natural environment. In this study, 33 wild populations of *Salicornia* were collected from different geographical areas around Urmia Lake during the flowering stage, and 55 morphological traits and 25 ISSR loci of the plant were analyzed. Based on morphological and molecular traits and the cluster analysis, *Salicornia* populations were divided into four and two groups, respectively.

**Results:**

Overall, the high percentage of polymorphic loci (65.69%), the average number of effective alleles per locus (1.63), and the Shannon data index (0.540) indicate that ISSR markers was used to identify genetic diversity. Molecular data cluster analysis divided the studied populations into two main groups, which included 12.12% and 87.88% of the populations, respectively. Based on the effective analysis of the population’s genetic structure and the precise classification of individuals into suitable sub-populations, the value of *K*=2 was calculated.

**Conclusions:**

The research findings indicated that the populations of *Salicornia* have a considerable diversity in morphological traits. Furthermore, markers UBC823, B, A7, and K, as well as markers with the Shannon index, effective allele, and large heterozygosis values, are the most effective markers in comparison with other markers used in this study. The findings of this study will aid in parental selection studies for breeding programs of *Salicornia* in future.

## Background

Genetic diversity in crops and orchards is an issue long considered by plant breeders searching for new sources of germplasm to perform gene transfer, phylogenetic testing, and marker selection, among other things [18].

Given the role of genetic diversity in advancing breeding programs and the importance of the local population, it is necessary to study the local population’s genetic diversity [31]. A variety of natural genetic resources in an area can provide beneficial genes for plant breeding. These genes have been formed and stored mainly in native plants for centuries [25]. Many of these native species have been being introduced as new plants due to their medicinal and industrial properties [11]. It is necessary to study genetic diversity among different species using morphological features to find desirable traits for further production [26]. Morphological traits obtained from visible mutations in morphology include a wide range of genes that control morphological characteristics based on the phenotypes and serve as the first markers. They used time immemorial, that is, the location of a gene chromosome determined [24]. The most treasured resources in any country include genetic resources. Plant stocks are used by breeders as a resource for genetic material for generating new varieties. For utilizing genetic resources at the highest efficiency, the stored genetic material should be known. Samples can be evaluated in accordance with the purpose of germplasm usage, including pathological, agronomic, morphological, biochemical, molecular, and histological dimensions. With the evaluation of germplasm, information about the weaknesses and strengths of the genotypes and populations and their potentials can be obtained, and genetic basis of each trait can be determined by these evaluations. Investigating genetic diversity in plants is significant from various dimensions. Generally, when genetic diversity is determined, it is beneficial for researchers for managing collections, conservation, maintenance, and specification of plants, as well as usage of plant collections [27]. The use of molecular markers in scientific research has opened up new possibilities for identifying and manipulating particular genes. Molecular markers have become increasingly important in evaluating species diversity and evolutionary relationships [15]. For researchers, the genetic analysis of plants is a foundation for characterizing natural plant genetic resource, detecting genetic diversity or genetic homogeneity, and selecting plants with specific traits such as the synthesis of desired chemicals and stress tolerance mechanisms [8]. *Salicornia* consists of approximately 15 genus and 68 species [29]. However, it is challenging to classify this plant species due to self-pollination and diversity in local populations.

Besides the loss of leaves and morphological identification indices and the small amount of dry matter compared to wet tissue, the accurate identification of species is difficult [3]. *Salicornia iranica* Akhani, an endemic species of *Salicornia* in Iran, grows in central Iran and is a diploid genus of *Salicornia* [1]. The habitats of this plant in Iran are Fars, Semnan, Gorgan, Bushehr, Hormozgan, Yazd, Khorasan, Khuzestan, Markazi, West and East Azerbaijan, Isfahan, Qom, and Tehran provinces [22]. According to studies, species collected from seven regions surrounding Urmia Lake have been identified as *Salicornia iranica* [22].

The *Salicornia* is important as a medicinal plant, and given the fact that there are not adequate and comprehensive studies in different fields of production. The current survey was conducted in order to (1) estimate the morphological and molecular variation among 33 wild *Salicornia* populations, (2) search for genetic structure of *Salicornia* populations and identify the most effective ISSR markers, and (3) identify the relationships between morphological characteristics and ISSR markers to partition the genetic variation within and among populations, and provide basic information for conservation and breeding programs. In this study, 33 populations of *Salicornia* grown around Urmia Lake were collected, and to evaluate the morphological and genetically diversity between different populations, 55 different morphological traits and 25 ISSR markers were studied; also, for future genetic modification and parent plant selection, the results can be made available to the breeders.

## Methods

**Fig. 1:**
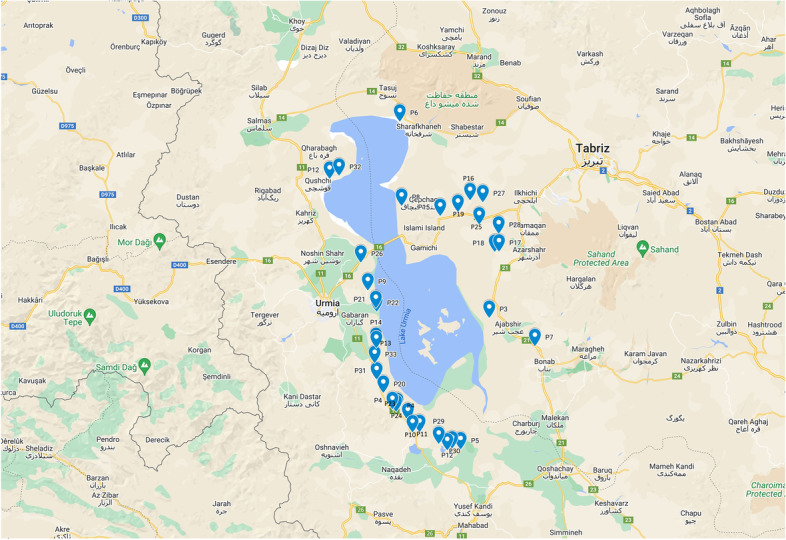
Sampling regions of different *Salicornia* populations around Urmia Lake. Map constructed with Google maps

In this study, 33 wild populations of *Salicornia* in full bloom and plant seeds were collected from different geographical areas in the lake’s vicinity (Table 1, Fig. 1). At the time of data collection, features such as the geographic area’s location and characteristics (altitude and latitude) were recorded. Some populations were geographically less than a few hundred meters apart, which were considered separately, based on field observations.

**Table 1:** Geographic characteristics of sampling regions of different *Salicornia* populations around Lake Urmia

Longitude	Latitude	Regions	Population	Code	Longitude	Latitude	Regions	Population	Code
45° 5′ 7.24″E	38° 0′ 2.55″N	West Azerbaijan	Qoshchi 1	P1	45° 50′ 23.82″E	37° 49′ 1.22″N	East Azerbaijan	Gogan khaslou II	P18
45° 5′ 7.24″E	38° 0′ 2.55″N	West Azerbaijan	Qoshchi 2	P2	45° 39′ 2.32″E	37° 52′ 34.46″N	East Azerbaijan	Saray Road	P19
45° 47′ 35.42″E	37° 30′ 27.75″N	East Azerbaijan	Port of Rahmanlu	P3	45° 21′ 50.18″E	37° 11′ 34.29″N	West Azerbaijan	Sand Plant	P20
45° 26′ 28.23″E	37° 8′ 31.49″N	West Azerbaijan	After medical sciences Univ. before Hasanlu dam	P4	45° 15′ 59.99″E	37° 31′ 35.60″N	West Azerbaijan	Isa- Can I	P21
45° 41′ 7.89″E	37° 2′ 9.74″N	West Azerbaijan	Dashkhaneh	P5	45° 15′ 59.99″E	37° 31′ 35.60″N	West Azerbaijan	Isa- Can II	P22
45° 28′ 21.76″E	38° 10′ 30.96″N	East Azerbaijan	Sharafkhaneh Port	P6	45° 26′ 51.08″E	37° 7′ 48.66″N	West Azerbaijan	Shirin-Bulagh I	P23
46° 0′ 31.73″E	37° 24′ 52.32″N	East Azerbaijan	Bonab plant	P7	45° 26′ 51.08″E	37° 7′ 48.66″N	West Azerbaijan	Shirin-Bulagh II	P24
45° 25′ 11.52″E	37° 54′ 15.67″N	East Azerbaijan	Islami Iceland	P8	45° 44′ 55.30″E	37° 52′ 2.41″N	East Azerbaijan	Aji Chai River	P25
45° 15′ 32.59″E	37° 35′ 15.63″N	West Azerbaijan	Chi-Chest	P9	45° 13′ 57.39″E	37° 43′ 9.29″N	West Azerbaijan	Road Police	P26
45° 28′ 49.08″E	37° 6′ 2.48″N	West Azerbaijan	Wetland in front of Hasanlu Dam I	P10	45° 45′ 16.60″E	37° 56′ 17.47″N	East Azerbaijan	Hassanabad River	P27
45° 28′ 49.08″E	37° 6′ 2.48″N	West Azerbaijan	Wetland in front of Hasanlu Dam II	P11	45° 49′ 23.52″E	37° 52′ 29.76″N	East Azerbaijan	Radio station	P28
45° 35′ 10.94″E	37° 2′ 39.24″N	West Azerbaijan	Solduz Wetland	P12	45° 37′ 34.52″E	37° 2′ 13.09″N	West Azerbaijan	Gerda- ghit I	P29
45° 17′ 17.52″E	37° 21′ 8.63″N	West Azerbaijan	Urmia Road Police	P13	45° 39′ 10.45″E	37° 1′ 53.30″N	West Azerbaijan	Gerda- ghit II	P30
45° 16′ 13.60″E	37° 22′ 34.67″N	West Azerbaijan	Before Urmia Road Police	P14	45° 19′ 32.19″E	37° 15′ 3.44″N	West Azerbaijan	Dizaj- dol	P31
45° 34′ 44.08″E	37° 51′ 55.05″N	East Azerbaijan	Saray	P15	45° 9′ 4.67″E	38° 1′ 9.86″N	West Azerbaijan	Mighatlou	P32
45° 42′ 7.39″E	37° 56′ 27.59″N	East Azerbaijan	Shekargah	P16	45° 18′ 19.35″E	37° 18′ 20.48″N	West Azerbaijan	Cement factory	P33
45° 50′ 23.82″E	37° 49′ 1.22″N	East Azerbaijan	Gogan khaslou I	P17

Fifty-five morphological traits were evaluated. Fifteen specimens were sampled per population, and for each plant, all 55 traits were calculated (Table 2). The morphological traits were measured in the Plant Physiology Laboratory, Horticulture Department, Faculty of Agriculture, Urmia University, and Herbarium, Faculty of Pharmacy, Tabriz University. The properties were measured using a ruler, digital caliper, scrubber, and optical microscope [12, 14].

**Table 2:** Morphological traits studied in *Salicornia* populations

Code	Traits	Measurement unit	Code	Traits	Measurement unit
V1	Height of plant from rooting point to apex	(cm)	V29	Number of sterile segments on the longest secondary	(Number)
V2	Stem diameter	(cm)	V30	Length of the longest tertiary branch	(cm)
V3	Height from rooting point to 1st branching point	(cm)	V31	Length of the terminal spike	(cm)
V4	Number of internodes	(Number)	V32	Number of fertile segments on terminal spike	(Number)
V5	Length of 1st internode	(cm)	V33	Number of sterile segments on terminal spike	(Number)
V6	Length of 2nd internode	(cm)	V34	Number of spike in 1st (basal) primary branch	(Number)
V7	Length of penultimate internode	(cm)	V35	Number of spike in penultimate branch	(Number)
V8	Length of ultimate internode	(cm)	V36	Number of spike in ultimate branch	(Number)
V9	Number of side primary branch	(Number)	V37	Height of 3rd fertile segment on terminal spike	(mm)
V10	Length of longest 1st (basal) primary branch	(cm)	V38	width of 3rd fertile segment on terminal spike	(mm)
V11	Average number of fertile segments on terminal spike in 1st primary branch	(Number)	V39	Height of central floret of 3rd fertile segment	(mm)
V12	Average number of sterile segments on terminal spike in 1st primary branch	(Number)	V40	Width of central floret of 3rd fertile segment	(mm)
V13	Length of longest 2nd primary branch	(cm)	V41	Height of side floret of 3rd fertile segment	(mm)
V14	Average number of fertile segments on terminal spike in 2nd primary branch	(Number)	V42	Width of side floret of 3rd fertile segment	(mm)
V15	Average number of sterile segments on terminal spike in 2nd primary branch	(Number)	V43	Width across apex of 3rd fertile segment	(mm)
V16	Length of the longest penultimate branch	(cm)	V44	Distance from tip of 3rd fertile segment to apex of middle floret	(mm)
V17	Number of fertile segments in penultimate branch	(Number)	V45	Distance between florets on 2nd fertile segment	(mm)
V18	Number of sterile segments in penultimate branch	(Number)	V46	Length of first sterile segment on terminal spike	(mm)
V19	Length of ultimate branch	(cm)	V47	Length of last sterile segment on terminal spike	(mm)
V20	Number of fertile segments in ultimate branch	(Number)	V48	Height of central seed	(mm)
V21	Number of sterile segments in ultimate branch	(Number)	V49	Width of central seed	(mm)
V22	Distance from apex to apex of ultimate branch	(cm)	V50	Height of side seed	(mm)
V23	Distance from apex to apex of 1st primary branch	(cm)	V51	Width of side seed	(mm)
V24	Number of secondary branches in 1st primary branch	(Number)	V52	Weight 1000 seed	(g)
V25	Number of secondary branches in 2nd primary branch	(Number)	V53	Length of Stomata	(μm)
V26	Maximum number of secondary on a primary branch	(Number)	V54	Width of Stomata	(μm)
V27	Length of longest secondary branch	(cm)	V55	Number of Stomata	(Number)
V28	Number of fertile segments on the longest secondary	(Number)			

### Studying genetic diversity

#### Molecular evaluation

CTAB approach [6] was used for extraction of individual genomic DNA. Spectrophotometry and 1% agarose gel electrophoresis were performed for evaluating the quantity and quality of the extracted DNA. Using 25 ISSR primers, genotypes were recorded in the subjects. Lodhi et al. [19] optimized PCR reactions and their temperature cycle. PCR was run in 15-μl reaction mixture, which consists of master mix 2× of 5 μl, primer 10pM of 1 μl, template DNA 50 ng/μl concentration of 2 μl, and sterile water of 7 μl. PCR amplification profile with 95 °C for 4 min of initial denaturation, followed by 30 cycles of 94 °C for 30 s, 41–58 °C for 1 min and 72 °C for 1 min, and followed by a final extension for 10 min at 72 °C. PCR amplicons were resolved on 0.8% agarose gel electrophoresis. Besides, using the GeneRuler’O Fermentas size indicator, the size of the band was determined.

The combination of markers was used for obtaining population structure according to the data by the use of STRUCTURE software 2.3.4 (30) with 50,000 MCMC repetitions and 50,000 in-Burn time in Admixture mode in varying values of *K* in a range of 1–20 (5 repetitions per *k*). This software was also used for estimating the membership share matrix (Q). With this matrix, it is shown that each member to what extent fits to the clusters. Using the same software, the average stabilization index (FST) was calculated for potential subgroups. The approach proposed by Evanno et al. [9] was used for determining the actual number of subpopulations. The basis of this approach is on Δ*K* statistic breaking a function’s slope when there is the maximum probability for a hypothetical number.

### Statistical analysis of data

The ANOVA and variation within-group were expressed as coefficient of variation for quantitative descriptors calculated for each group and the whole collection. Principal components analysis (PCA) was performed using XLSTAT 2018.1 statistical software. The first and second principal component axes scores were plotted to aid visualization of origin group differences and detect morphological variation in the collection.

#### Analysis of data

Population structure was studied using bands from all marker matrices. Using different algorithms, such as UPGMA, single linkage, and complete linkage, cluster analysis was performed. These algorithms were employed as zero (absence) and one (presence) scoring. The clusters were drawn in the present work using Mega software. Also other data were analyzed using the following software: NTSYSpc version 2.0.1.5, SAS 9.2 (ANOVA analysis), SPSS (means), Mega (Molecular analysis), and PopGene (Molecular analysis).

## Results

The variation and the mean traits were examined for different populations. Among the studied populations of *Salicornia*, the non-fertile parts on the longest secondary branch (V29) (84.75%), the fertile parts on the longest secondary branch (V28) (81.49%), and the flowering plants in the first lateral branch (V34) (66.13%) had the highest diversity (Table 3). According to the results, the highest and lowest number of primary lateral branches (V9) was observed in P27, 43, and P22, 13.4, respectively. Complete information about other variables is given in Table 3.

**Table 3:** Descriptive statistics for the estimated morphological traits studied in *Salicornia* populations

Variable	CV	SD	Mean	Max	Min	Variable	CV	SD	Mean	Max	Min
**V1**	20.65	6.73	32.59	50.12	23.70	**V28**	81.49	284.65	349.32	1473.2	8.80
**V2**	21.47	0.16	0.76	1.23	0.41	**V29**	84.75	90.78	107.12	444.40	2.40
**V3**	76.91	0.98	1.28	6.38	0.56	**V30**	37.36	3.16	8.45	15.60	0.10
**V4**	23.16	4.73	20.42	29.40	12.00	**V31**	43.03	2.16	5.02	9.08	0.94
**V5**	18.87	0.22	1.15	1.55	0.59	**V32**	39.66	6.75	17.01	30.60	3.20
**V6**	18.17	0.25	1.36	1.85	0.85	**V33**	26.65	0.61	2.29	4.60	1.80
**V7**	20.73	0.23	1.09	1.48	0.72	**V34**	66.13	99.76	150.84	366.80	18.4
**V8**	21.99	0.21	0.94	1.36	0.62	**V35**	40.55	2.94	7.25	12.80	3.00
**V9**	24.74	7.02	28.36	43.00	13.40	**V36**	44.93	2.44	5.42	14.60	2.60
**V10**	24.87	6.57	26.41	41.00	15.10	**V37**	16.05	0.56	3.47	4.86	2.38
**V11**	37.90	5.49	14.48	28.70	4.60	**V38**	21.13	0.60	2.84	4.30	1.64
**V12**	9.97	0.21	2.13	2.90	2.00	**V39**	18.23	0.46	2.53	3.33	1.52
**V13**	25.98	6.27	24.13	37.46	13.28	**V40**	17.20	0.36	2.11	2.90	1.23
**V14**	37.27	5.42	14.54	28.76	4.60	**V41**	22.00	0.33	1.50	2.20	0.83
**V15**	11.90	0.26	2.17	2.84	2.00	**V42**	25.80	0.38	1.46	2.57	0.87
**V16**	31.41	2.25	7.16	11.92	3.34	**V43**	27.52	0.13	0.46	0.93	0.21
**V17**	54.55	36.7	67.32	154.0	20.20	**V44**	32.26	0.18	0.55	1.01	0.33
**V18**	48.81	7.46	15.28	36.40	6.40	**V45**	18.59	0.62	3.35	5.03	2.03
**V19**	29.49	1.77	5.99	9.72	2.60	**V46**	28.39	0.62	2.18	3.53	1.05
**V20**	47.00	23.6	50.38	112.0	17.00	**V47**	29.81	0.33	1.11	1.89	0.73
**V21**	44.66	4.79	10.73	27.60	4.60	**V48**	10.88	0.20	1.87	2.27	1.24
**V22**	47.83	6.44	13.46	43.00	5.64	**V49**	13.09	0.13	0.96	1.27	0.70
**V23**	31.19	14.4	46.34	77.50	9.76	**V50**	14.37	0.23	1.58	2.11	1.19
**V24**	34.44	6.11	17.73	29.60	6.20	**V51**	19.25	0.16	0.84	1.47	0.57
**V25**	33.77	6.24	18.48	33.40	7.60	**V52**	31.21	0.12	0.38	0.75	0.22
**V26**	32.15	7.24	22.50	39.00	9.20	**V53**	17.78	2.75	15.47	19.40	7.00
**V27**	31.57	5.59	17.71	27.94	2.74	**V54**	22.40	1.93	8.61	16.40	5.70
						**V55**	21.69	1.52	6.99	11.00	4.60

**Fig. 2:**
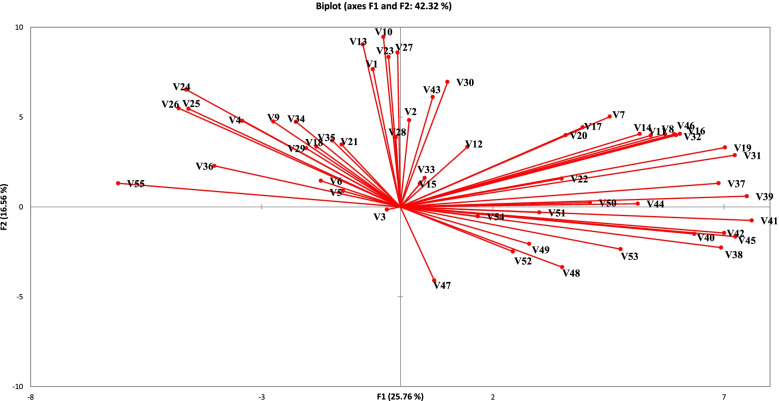
Plot distribution of 33 *Salicornia* populations and studied traits depending on principal component axes PC1 and PC2. The first two components had a variation of (1) 25.76% and (2) 16.56%

The first five of the 32 principal components (PCs) obtained have eigenvalues greater than 2. Together, they accounted for about 67.28% of the total variance of *Salicornia* traits (Fig. 2, Table 4). The first two PCs account for 42.32% of the total variability (25.76% and 16.56%, respectively) (Tables 4 and 5). PC1 represent ration of V7, V8, V11, V14, V16, V19, V25, V26, V31, V32, V37, V38, V39, V40, V41, V42, V44, V45, V46, V53, and V55. PC2 describe the ration of V1, V10, V13, V23, V24, V27, V30, and V43. Figure 2 and Table 4 show that traits lie around PC1 and PC2 center. The large variability of the traits allows observation such as V10, V31, V39, V41, and V45, where the amount of length of longest 1st primary branch, length of the terminal spike, height of central floret of 3rd fertile segment, height of side floret of 3rd fertile segment, and distance between florets on 2nd fertile segment.

**Table 4:** Principal components for studied morphological traits in *Salicornia* populations

Variable	F1	F2	F3	F4	F5	Variable	F1	F2	F3	F4	F5
V1	0.005	0.546*	0.021	0.314	0.002	V30	0.015	0.449*	0.011	0.254	0
V2	0.001	0.216	0.05	0.253*	0.075	V31	0.758*	0.077	0.016	0.045	0.005
V3	0.001	0	0.224	0.069	0.006	V32	0.515*	0.148	0.045	0.112	0.016
V4	0.169	0.213	0.041	0.400*	0.013	V33	0.004	0.024	0.096	0.122	0.051
V5	0.022	0.007	0.123	0.042	0.505*	V34	0.074	0.208	0.049	0.350*	0.026
V6	0.043	0.02	0.006	0.04	0.592*	V35	0.031	0.127	0.488*	0.101	0.02
V7	0.297*	0.235	0.004	0.048	0	V36	0.234	0.048	0.358*	0.054	0.056
V8	0.475*	0.152	0.061	0.001	0.009	V37	0.686*	0.016	0.001	0.003	0.016
V9	0.109	0.21	0.065	0.313*	0.071	V38	0.697*	0.048	0.051	0	0.009
V10	0.002	0.831*	0.093	0.01	0.004	V39	0.813*	0.003	0.001	0.001	0.004
V11	0.425*	0.147	0.142	0.01	0.048	V40	0.585*	0.021	0.059	0.003	0.002
V12	0.03	0.103	0.374*	0.104	0.14	V41	0.836*	0.005	0.001	0	0.001
V13	0.009	0.759*	0.113	0.002	0.008	V42	0.710*	0.02	0.021	0.001	0
V14	0.388*	0.153	0.147	0.03	0.067	V43	0.007	0.347*	0.004	0.041	0.002
V15	0.003	0.015	0.302*	0.09	0.193	V44	0.382*	0	0.062	0.035	0.08
V16	0.529*	0.154	0.181	0	0.003	V45	0.760*	0.026	0.017	0.002	0.018
V17	0.225	0.183	0.402*	0.016	0.004	V46	0.508*	0.152	0.016	0.007	0.02
V18	0.048	0.105	0.587*	0.134	0.04	V47	0.008	0.156	0.072	0.017	0.003
V19	0.714*	0.101	0.088	0.012	0	V48	0.178	0.105	0	0.003	0.07
V20	0.185	0.148	0.366*	0.001	0	V49	0.113	0.04	0.074	0.02	0.02
V21	0.023	0.112	0.561*	0.001	0	V50	0.244	0.001	0.008	0.132	0.037
V22	0.176	0.023	0.169	0.081	0.005	V51	0.131	0.001	0.023	0.123	0.02
V23	0.001	0.646*	0.164	0.016	0.009	V52	0.086	0.058	0.003	0.221*	0.181
V24	0.31	0.395*	0.014	0.002	0.002	V53	0.329*	0.052	0.034	0.001	0.001
V25	0.304*	0.277	0.162	0.003	0	V54	0.041	0.003	0.123	0.041	0.033
V26	0.333*	0.28	0.012	0.049	0.004	V55	0.539*	0.016	0.072	0.005	0.001
V27	0	0.686*	0.046	0.077	0.005						
V28	0	0.139	0.1	0.344*	0.175						
V29	0.06	0.099	0.107	0.366*	0.092						

*Significant at *P* < 0.05

**Table 5:** Eigenvalue, proportion, and cumulative variation of analyzed components

	F1	F3	F3	F4	F5
Eigenvalue	14.17	9.11	6.43	4.53	2.77
Variances	25.76	16.56	11.7	8.23	5.03
Cumulative variances	25.76	42.32	54.02	62.24	67.28

**Fig. 3:**
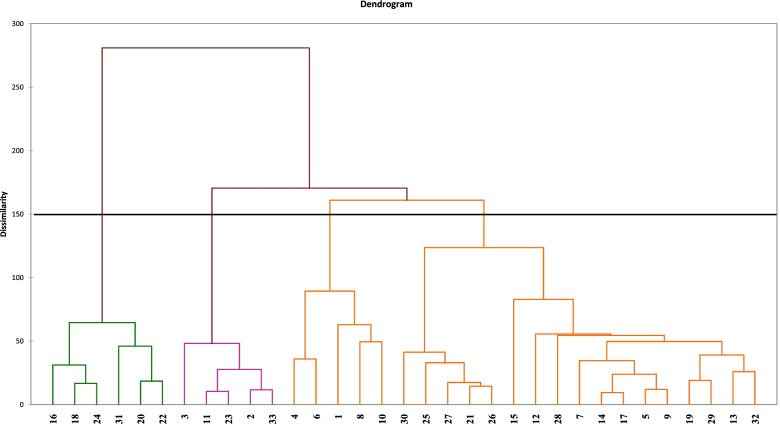
Hierarchical cluster analysis (HCA) of different *Salicornia* populations based on 55 main morphologic traits

According to the morphological traits results of cluster analyses by the Ward method, *Salicornia* populations were assigned to four groups (Fig. 3). The first group contained 8.18% of populations (P16, P18, P24, P31, P20, and P22). In this group, populations with a short height, long spike, greater weight of 1000 seed, low number of stomata, and the width across the apex on the third fertile segment were more abundant than other populations. The morphotype and inflorescences of this group were distinct from other groups. The second group covered 15.15% of the whole population (P3, P11, P23, P2, and P33), comprising populations that were within the average range of sizes for diverse traits. The third group hosted 15.15% of the population (P4, P6, P1, P8, and P10), and the fourth group included 51.51% of the population (P9, P30, P25, P27, P21, P26, P15, P12, P28, P7, P14, P17, P5, P19, P29, P13, and p32). These populations had a great height, more internodes, more lateral branches, more stomata, a great weight of 1000 seeds, and the width of the third fertile segment on the terminal spike. The accurate number of groups was identified using the detection function.

### Genetic diversity of *Salicornia* populations

**Fig. 4:**
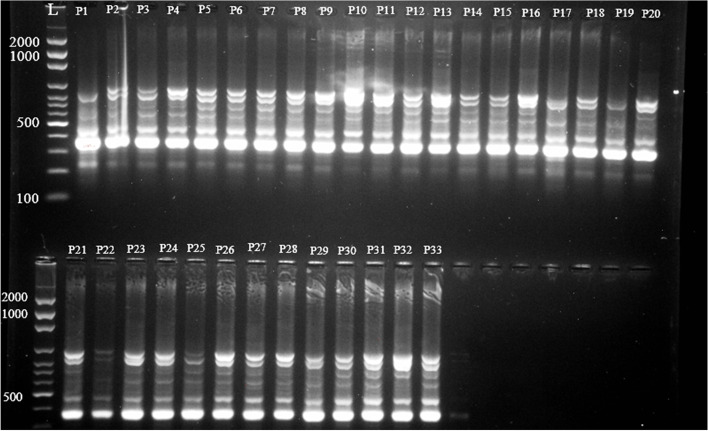
Fingerprint images of Salicornia populations with primer UBC860

We evaluated genetic diversity in 33 *Salicornia* populations using 42 ISSR primers. Twenty-three primers out of 42 primers under study generated a polymorphic band design at the suitable resolution, which were employed for the subsequent analysis phases (Table 6). In total, 204 alleles with an average 8.87 allele per marker were detected, 134 of them were polymorphic (65.69%). The ratio of markers to primer was 1 to 14, averagely 5.82 (Table 6, Fig. 4).

**Table 6:** Markers name, annealing temperature (°C), total band, polymorphic band, and percent of polymorphic band

	Markers’ name	Primer sequences	Annealing temperature	Total bond	Polymorphic band	Polymorphic band percent
1	A	CACACACACACAGG	44 °C	10	8	80
2	B	CACACACACACAAC	41 °C	11	6	54.55
3	F	GAGAGAGAGAGAGG	44 °C	8	3	37.50
4	G	GTGGTGGTGGTGCC	44 °C	10	4	40
5	H	AGAAGAAGAGAGGAGGT	50 °C	4	2	50
6	I	AGAAGAAGAGAGGAGGC	52 °C	5	4	80
7	J	ACAACAACACACCACCT	50 °C	10	9	90
8	K	ACAACAACACACCACCG	52 °C	10	6	60
9	A7	AGAGAGAGAGAGAGAGAGAGT	58 °C	10	5	50
10	A12	AGAGAGAGAGAGCC	52 °C	9	3	33.33
11	A13	GTGTGTGTGTGTCC	55 °C	14	13	92.86
12	UBC818	CACACACACACACACAG	56 °C	5	2	40
13	UBC825	ACACACACACACACACT	55 °C	7	6	85.71
14	UBC849	GTGTGTGTGTGTGTGTCG	55 °C	3	1	33.33
15	UBC811	GAGAGAGAGAGAGAGAC	54 °C	11	8	72.73
16	UBC844	CTCTCTCTCTCTCTCTRC	56 °C	9	6	66.67
17	UBC823	TCTCTCTCTCTCTCTCCC	48.5 °C	4	3	75
18	UBC834	CTCTCTCTCTCTCTCTAC	51.3 °C	17	14	82.35
19	UBC850	GTGTGTGTGTGTGTGTGTGTGTGTGTGTGC	56.03 °C	12	11	91.67
20	UBC860	TGTGTGTGTGTGTA	40 °C	6	3	50
21	201274	CACACACACACARY	42 °C	14	11	78.57
22	201275	CACACACACACARG	43 °C	6	3	50
23	201246	AGAGAGAGAGAGAGYC	47 °C	9	3	33.33
	Total			204	134	65.69

The number of effective (Ne) alleles ranged from 1.25 for UBC849 and 1.92 for in PB with an average 1.63 per locus. Maximum value of this statistic shows that alleles have identical frequency in this location, and this statistic’s minimum shows the rarity of other alleles and one allele’s high frequency in samples.

In investigating allelic diversity, the highest observed heterozygosity was found by B marker with 0.477; however, the lowest observed heterozygosity was noticed by UBC849 marker with 0.199. Besides, the highest expected heterozygosity was observed at approximately 0.484 by B marker, and the lowest expected heterozygosity was observed at approximately 0.203 in the UBC849 marker. Examining Shannon index (I) values showed that the highest value for this index was in marker B with a 0.670 and the lowest value was in UBC849 marker as 0.351 (Table 7).

**Table 7:** Number of effective alleles (Ne), Shannon index (I), expected heterozygosity (He), and observed heterozygosity (Ho)

Primer	Ne	I	He	Ho
201274	1.665	0.573	0.388	0.394
p825-1	1.602	0.526	0.353	0.358
A13	1.654	0.564	0.380	0.386
I	1.725	0.595	0.409	0.415
UBC811	1.724	0.599	0.411	0.418
UBC823	1.819	0.640	0.448	0.456
A7	1.805	0.624	0.435	0.442
UBC850	1.673	0.574	0.390	0.396
UBC849	1.249	0.351	0.199	0.203
UBC860	1.654	0.574	0.388	0.394
A	1.631	0.559	0.375	0.381
B	1.917	0.670	0.477	0.484
F	1.494	0.446	0.290	0.295
G	1.406	0.391	0.247	0.251
H	1.711	0.602	0.413	0.419
J	1.483	0.463	0.299	0.303
K	1.571	0.518	0.343	0.348
UBC834	1.788	0.620	0.431	0.438
UBC844	1.501	0.474	0.307	0.312
UBC818	1.558	0.492	0.324	0.329
201275	1.422	0.396	0.257	0.261
201246	1.766	0.620	0.429	0.436
Mean	1.628	0.540	0.378	0.384

**Fig. 5:**
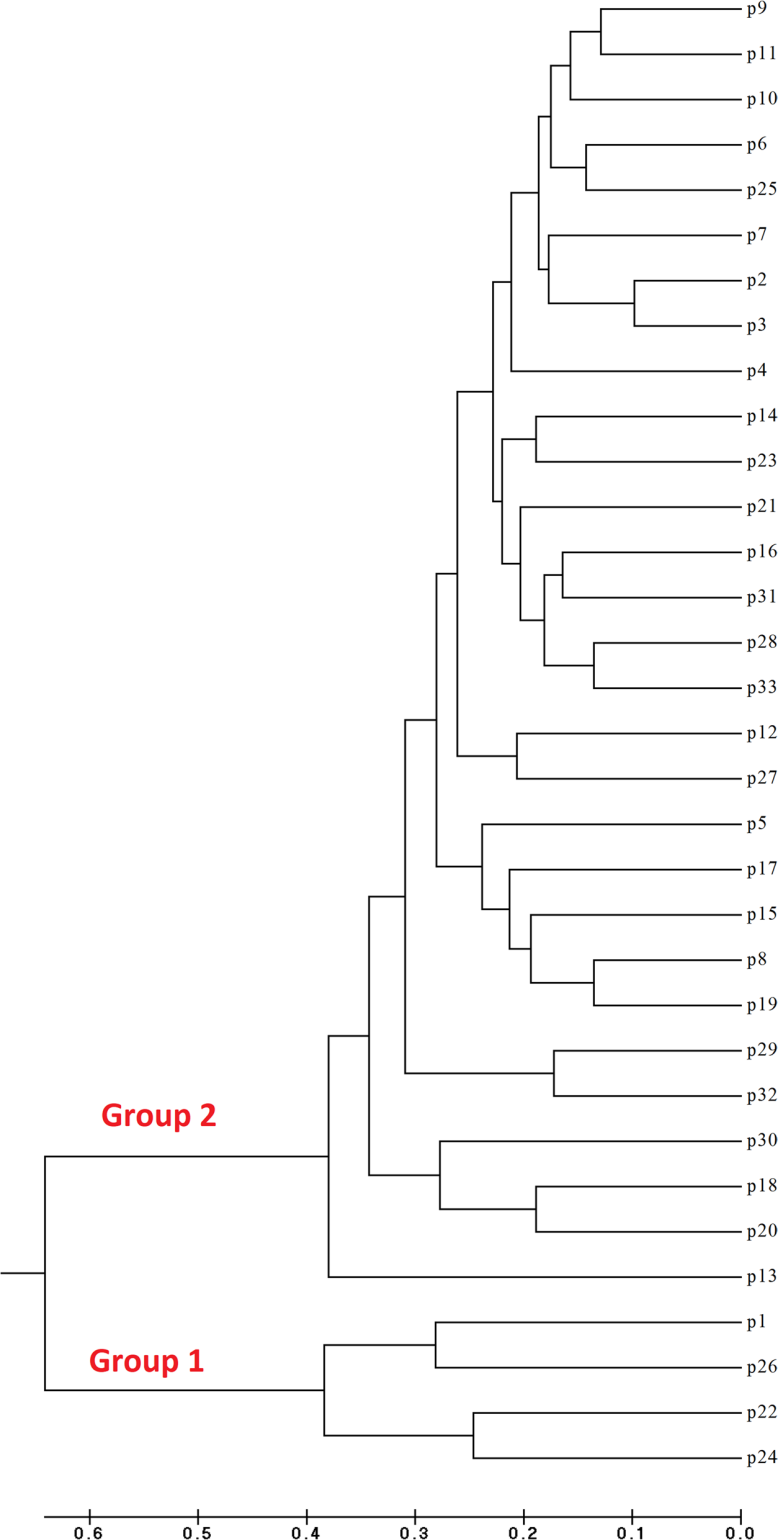
Dendrogram showing relationships among 33 population of *Salicornia*. Group I represents the four populations while group II represents other 29 populations based on ISSR marker (algorithm UPGM)

The Jaccard similarity coefficient and UPGMA algorithm were used for dividing different populations into two separate groups. The first group contained 12.12% and the second group included 87.88% of the masses. Two subgroups were made in the first group, which the first one included P24, P22, P26, and P1. The second group contains the residual 29 populations (P13, P20, P18, P30, P32, P29, P19, P8, P15, P17, P5, P27, P12, P33, P28, P31, P16, P21, P23, P14, P4, P3, P2, P7, P25, P6, P10, P11, P9), which was classified into two subgroups. The first one is composed of just the P13 population. Also, this population was approximately different from other ones (Fig. 5).

Structure 2.3.1 software was used for analyzing genetic population structure and precise classifying individuals into proper subpopulations. As shown by a two-way diagram of optimal determination of K with ISSR indicator, the ISSR primer shows the best *K* as 2, i.e., two subpopulations (*K* = 2) in the cultivars under study. The group was specified (Tables 8 and 9).

**Table 8:** Mean Stabilization Index (FST) of each cluster based on cluster analysis based on Bayesian model at *K* = 2

Cluster	FST
I	0.0003
II	0.2156

**Table 9:** Population membership matrix in each cluster based on Structure 2.3.1 software calculations at *K* = 2

Population	Group 1	Group 2	Population	Group 1	Group 2
P1	0.007	0.993	P18	0.991	0.009
P2	0.986	0.014	P19	0.196	0.804
P3	0.985	0.015	P20	0.995	0.005
P4	0.996	0.004	P21	0.010	0.990
P5	0.953	0.047	P22	0.571	0.429
P6	0.992	0.008	P23	0.992	0.008
P7	0.990	0.010	P24	0.008	0.992
P8	0.921	0.079	P25	0.995	0.005
P9	0.994	0.006	P26	0.579	0.421
P10	0.989	0.011	P27	0.956	0.044
P11	0.988	0.012	P28	0.992	0.008
P12	0.993	0.007	P29	0.990	0.010
P13	0.991	0.009	P30	0.985	0.015
P14	0.992	0.008	P31	0.988	0.012
P15	0.140	0.860	P32	0.994	0.006
P16	0.983	0.017	P33	0.902	0.098
P17	0.041	0.959			

**Fig. 6:**
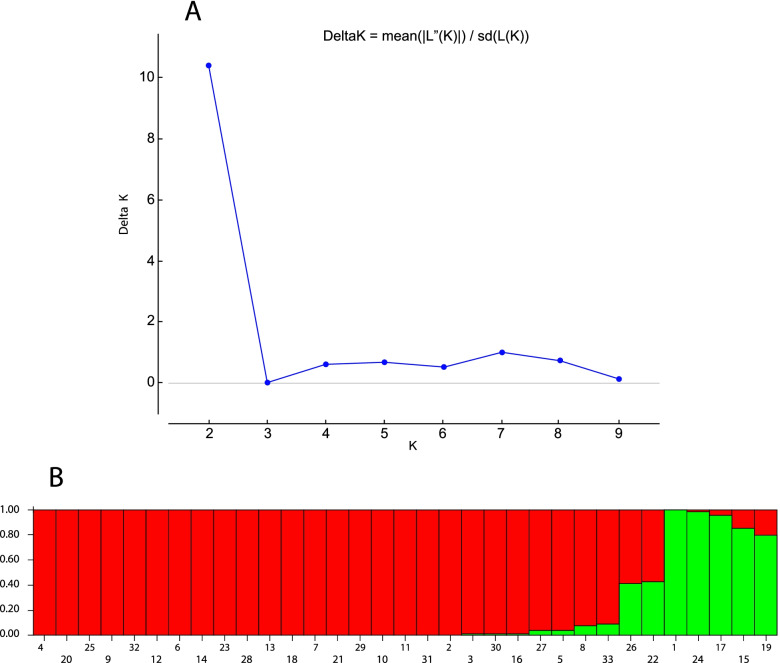
Population genetic structure of 33 populations of *Salicornia*. **A** Graph showing the best value of *K*=2. **B** Bar plot of structure at *K*=2 indicating less admixture between analyzed populations. Each color represents a subset or cluster. The vertical axis shows the coefficient of belonging of each person to each cluster

The stabilization index (FST) is a common and appropriate measure for genetic differentiation among groups and populations. When the FST is higher, a better allele differentiation is obtained, with a higher allele stabilization rate. Potential subgroups in *K* = 2 show the difference among the populations under study in two potential groups. Besides, the individuals’ matrix of the share in these groups (Tables 4 and 5) indicated belonging populations with high coefficients to one group. Bar plot results demonstrated inclusion of 26 *Salicornia* populations in the first group (red) and 5 populations in the second group (green), with 2 populations had a complex structure (Fig. 6).

## Discussion

The results showed that there was a significant difference between the studied populations in terms of traits in the question. Based on the mean of traits measured in the population, traits with a high percentage of variance had a wide range of trait quantities and offered a more extensive choice for traits. This difference is due to the impact of both genetic and environmental factors. Studies have shown that fluctuations in soil and water salinity lead to physiological and phenotypic changes in the plant. Also, high plant density in a population restricts the number of branches and glaciers formed in the plant [13]. Self-pollination in plants, especially in diploid species, due to the flower’s unique structure, leads to the formation of various local populations in *Salicornia* [5]. The phenotypic variation coefficient between traits results in morphologically different plants manifests distinct genetic variations in different regions [28]. Together with the weight of 1000 seeds, these traits undermine the plants’ ability to produce satisfying seeds. With an increase in the number of internodes and lateral branches, the weight of 1000 seeds drops. Most of the plant energy comes spent on vegetative growth. Studies have focused on *Salicornia*’s two species in Iran (*S. Biglovi* and *Salicornia persica*). In *S. biglawi* species, raising the salinity of irrigation water to 45 dS/m reduces the height and dry weight of the plant. In *Persica* species, increasing the irrigation water salinity had no effect on plant height but significantly decreased the dry weight [28].

The cluster analysis results showed that (Fig. 3) the clustering of populations is incompatible with geographical distribution. It may be due to sources of seed diversity caused by migration to different areas. Therefore, it may not be limited to different geographical regions in selecting parents for breeding projects, but it should be consistent with each population’s specific capacities. By studying *Salicornia pusilla*, researchers have found that the plant seeds remain attached to the inflorescence after ripening, and the spikes are trapped by a separating layer of the plant isolated in the water that may keep moving with the flow of water up to 3 months. They may even germinate but do not grow until the seeds are deposited in sediments [4]. This feature may explain the common seed origin in the studied populations. Using 22 growth parameters, the researchers evaluated 11 *S. bigelovii* populations in the field and divided the cluster analysis of studied populations into four groups [20]. Contrary to our study, the results of research on the genetic diversity of six *Salicornia ramosissima* populations in central Germany showed that it is consistent with geographical distribution [17]. A review of the genetic diversity of the two species of saline *Salsola* manifested a significant difference in this plant and the environmental conditions of the plant, suggesting that disparity in salinity, nutrition, pH, and soil moisture changes the vegetative type of plants [30]. The results of analyzing the main components confirmed the clustering obtained from the cluster decomposition. The analysis of main components sheds light on the difference between individuals and allowing the identification of groups and the relationship between individuals and variables [21]. Based on the results of this analysis and multivariate analysis, four *Salicornia* populations were divided into three separate groups: the first component (46.11%), the second component (41.35%), and the overall component (87.46%) of the entire diversity. The study of the morphological diversity patterns of 52 *Salicornia* populations in 31 regions of Northern Europe using 28 morphological traits demonstrated diversity in the studied populations. The main components’ analysis revealed the first five components accounted for 79.8% of the total diversity. In the first component, characteristic spikes included the length of fertile segment and length of the spike (explaining 40% of the diversity, and in the second component, they included the size of the plant and the branches (explaining18.1% of the diversity) [10]. The findings are aligned with our results. Principal component analysis revealed that V7, V8, V11, V14, V16, V19, V25, V26, V31, V32, V37, V38, V39, V40, V41, V42, V44, V45, V46, V53, and V55 contributed mostly to diversity.

Though the association between regional diversity was not that evident, a close look at the scatter plot revealed some regional adaptation level was observed. Such regional variability could be due to geographic isolation and microclimatic differences between regions. Factors such as plant population isolation, adaptation to the environment due to declining lake water levels, and strong self-pollination within the plant population may contribute to *Salicornia*’s population diversity. The degree of morphological differentials is significantly noticeable in different populations from four groups.

The research findings indicated that markers UBC823, B, A7, and K, and with the Shannon index, effective allele, and large heterozygosity values, are markers with the highest effectiveness compared to other markers utilized, and they are used better than other compounds in genetic distance.

As stated by Dirlewanger et al. [7], there is a relationship between the alleles number in each gene locus and the number of used markers and the samples’ number. According to the findings of research on the genetic diversity of six populations of *Salicornia herbacea* in South Korea, where 6 ISSR markers were used, 39 polymorphic bands were obtained out of 49 bands, with an average of the effective allele for each gene locus as 1.22. The mean genetic index was 0.249 and the mean Shannon index was 0.382. These researchers mentioned that for achieving high diversity in populations *Salicornia*, a wider research scope is required to be chosen [16]. Using ISSR markers to identify genotypic differences among the 23 genotypes of finger millet revealed a high degree of polymorphism supported by substantial differences in all marker parameters [33].

These populations were separately gathered because of varying morphological types compared to other populations. Also, this difference is shown in the results. The second subgroup included P26 and P1 populations, with different appearances compared to other populations. They had a taller plant than average, particularly the taller plant was observed in P26 population among all populations. Moreover, long glazes were observed in these two populations. Additionally, it shows all botanical properties of *S. Iranica* [1].

In earlier Iranian research works on *Salicornia*, 36 samples of *Salicornia* were collected by Heydarian [10] from different saline areas. He specified this plant’s genetic diversity by the use of 17 RAPD markers, Jaccard similarity coefficient, and UPGMA approach. The subjects were categorized into 7 classes. Moreover, 18 *Salicornia* populations were evaluated by Mohammadi [23], which were collected from different regions in Iran. He used AFLP markers and categorized the individuals into 4 groups by the use of UPGMA method and Jaccard similarity coefficient. As shown by the research in this work, the researcher collected species from 7 regions near Lake Urmia and *S. iranica* are presented, all in a group. In this research, *S. iranica* species were separated from *S. persica* species using the AFLP marker, and they were placed in a subgroup. Additionally, the populations gathered from each area were put in a different subgroup. The genetic diversity in 11 *Salicornia brachiata* populations was evaluated in India using 15 ISSR and 15 RAPD primers [2]. The investigated populations showed high diversity. It was also observed in both markers of the populations under study. They were grouped into 3 groups.

The resulting bar plot showed that when the membership percentage to a cluster for a genotype is higher than or equal to 0.7, the genotype is allocated to that cluster, while if the percentage is below it, it is considered as a mixed genotype (hybrid) [32]. Generally, when the average effective allele numbers per gene locus (1.63), the polymorphic gene loci percentage (65.69%), and the Shannon data index (0.540) are high, it is indicated that we can use ISSR markers for identifying genetic diversity.

## Conclusions

This study showed that *Salicornia* populations growing around Urmia Lake had considerable diversity in morphological and ISSR characteristics. The incompatibility of population clustering with their geographical distribution may be due to different populations’ exact seed origins. The populations under the genetic study were divided into two major groups based on marker data, including 12.12% and 87.88%. The *K* value was obtained as two according to the practical analysis of the population’s genetic structure and the accurate individuals’ classification to suitable sub-populations. The populations under study were classified into two groups because *Salicornia* is a self-pollinated plant. Differences in morphological and genetic grouping may be due to the environment’s effect on morphological traits, while in genetic traits, the difference between the populations may be due to the populations’ isolation due to the lowering of the lake water, and the plant was directed towards self-breeding. Combining morphological and ISSR data may be more effective for defining genetic variation and genetic diversity within the *Salicornia* population.

## Data Availability

All data is provided in full in the results section of this paper. Expression all morphological and molecular data is openly available from Dryad at 10.5061/dryad.83bk3j9r

## Abbreviations

ISSR: Inter Simple Sequence Repeat
CTAB: Cetyltrimethylammonium bromide
MCMC: Markov Chain Monte Carlo
UPGMA: Unweight Pair Group Method with Arithmetic Mean
PCA: Principal component analysis
